# Chicago Medical Society

**Published:** 1888-09

**Authors:** 


REGULAR MEETING, May 21, 1888.

Dr. B. M. Behrens read a paper entitled "Cutting on the Drumhead," which was interesting and suggestive in its matter. It was a plea for instrumental interference in the cases of middle-ear disease which do not yield to what the writer calls uninstrumental methods—inflation or catheterization included. He describes his own method as follows:

"Instead of giving the indications for a probing I shall explain to my professional brethren how I proceed after having treated a case with the common non-instrumental remedies without any benefit. After a thorough cleaning of the meatus I apply a 4 per cent. solution of cocaine, and then make a longitudinal cut with the lancet in the upper part of the drumhead, back of the malleus and beneath the ligamentum posterius, so that I can easily reach the hearing bones. With the probe I examine the mobility of the stapes, or if I am not satisfied with probing, I apply the hook. Usually this will give me an idea about it. If there are striated lines in the drumhead, that seems to indicate adhesion in the middle ear. I replace the hook with a common straight convex ear-knife and divide them. We find striated lines in the drumhead in different conditions of the ear, from the best hearing to complete deafness, without knowing their importance in every individual case, if we don't, divide them where we are in doubt. Has no benefit been derived from this proceeding, we can stop, or we can go further. In the first place I simply apply some iodoformed cotton-batting, and the cut heals up again in a couple of days without the least damage being done. In the second place I apply more cocaine, and with a little sulphuric acid on the probe I cauterize it. After a few days I then have got an opening large enough to allow me to inject the internal wall of the middle ear to make mobilization trials, and, in case I find it necessary, to cut the tensor tympanic or stapes muscle—in short, to increase my diagnostic and therapeutic apparatus to an extent that can only benefit the otologist and never harm the patient; that is, at any rate, my experience. As to the question, how long the mobilization trials are going to be made, I have found it of most benefit to employ very little force, and for only a couple of seconds. By applying iodoformed cotton-batting from day to day, this proceeding can be repeated often, and if the cut tends to closing a new application of sulphuric acid can be made to prevent it. For how many days it is to be continued, it is not easy to tell. I usually do it four to eight days, according to the severity of the symptoms on account of irritation, tenderness, and the opening of the drumhead; any control as to improvement of hearing or abatement of noises is not easy to make, so I simply apply the iodoformed cotton, and let the wound heal up again. First, eight to fourteen days after I can make any examination as to the benefit derived from my proceeding. The result will indicate whether the process is to be repeated or not."

After a discussion of the history of the procedure, and a description of the various ways in which it has been employed, the author continues: "At last an appeal to my colleagues. I don't expect that any otologist shall jump at this proceeding of mine and take it for granted that this is the way, that after Urbautschitsch may lead to a mighty elevation of the otiatric, but shall only ask them to give it an honest trial in all cases of affection of the middle ear that are not benefited by our usual non-instrumental remedies. As any new idea, it has to be worked up, and the satisfaction it has given me in the last year entitles me to lay it before the medical profession and claim for it the right of being given a trial, the more as it has never done any harm. If we can but save a few of our fellow-beings from utter deafness by a method different from the usual way of treating with Politzer or catheter, where these are insufficient, we shall have more regard for this branch of the medical science and feel proud of having achieved a little more than what every non-expert may happen to achieve.

As the treatment of catarrh of the middle ear stands to-day, the pessimism is explicable, and no wonder that the soft methods have so many adherents as they have. Of those cases that I have treated with my proceeding, some few are selected that, given in detail, will, it is to be hoped, encourage my professional brethren to adopt it. Of course it will often fail to be of any benefit, but I have also pretended only, that it has given me more satisfaction than any other proceeding."

The author reported four cases in explanation, and closed with the following remark: "My sincere hope is that these cases, especially the last one, will do away with the pessimism to some extent, and deprive the 'soft methods' of some of its adherents."

The discussion of the paper developed nothing not fully covered by the paper itself.

EXHIBITION OF SPECIMENS.

Dr. T. B. Swartz exhibited a uterus, removed per vaginam for carcinoma of the cervix, and a dermoid cyst of the left ovary. The first case was one of interest, for several reasons. The diagnosis was confirmed by competent microscopical examination. The recovery at that time, four weeks subsequent to the operation, seemed complete. The patient was thirty-eight years old, had borne three children. She entered St. Luke's Hospital on May 3, and on May 5 Professor Dudley performed vaginal hysterectomy. The uterus was pulled down by means of forceps, the posterior cul-de-sac was entered, the entire uterus girdled, and the right broad ligament secured by hæmostatic pressure forceps and severed. The uterus then was turned out of the vagina and the left broad ligament served the same way. The operation was completed in twelve minutes. The hæmorrhage was controlled by hæmostatic forceps. The peritoneum was brought together by means of small pressure forceps; no sutures and no ligatures were used. Some forceps were removed in twenty-four hours, others in forty-eight hours, and those clamping the broad ligaments were removed in seventy-two hours.

The dermoid cyst of the left ovary was also removed by Professor Dudley at St. Luke's Hospital. The history of the case presented no points of special interest.

Dr. Henry T. Byford exhibited the ovaries which he had removed that morning from a virgin, in a case of a dysmenorrhœa which had resisted all other means of cure. He referred to a similar case in which he removed the ovaries one year ago, in which the operation was followed by complete recovery from the dysmenorrhœa.

